# Supplementary material for: FunPred 3.0: improved protein function prediction using protein interaction network
Source: PeerJ. 2019 May 22;7:e6830. doi: 10.7717/peerj.6830 (PMC6535044; doi:10.7717/peerj.6830)
Supplement: Supplemental Information 1 — Information about Protein-Protein interaction network, Protein pair function prediction, MIPS database and physico-chemical properties. [file peerj-07-6830-s001.docx]

**FunPred 3.0: Improved Protein function prediction using protein interaction network: Supplementary Document**

Sovan Saha^1^, Piyali Chatterjee^2^, Subhadip Basu^3^, Mita Nasipuri^3^, Dariusz Plewczynski ^4,5^

^1^ Department of Computer Science and Engineering, Dr. Sudhir Chandra Sur Degree Engineering College, DumDum, Kolkata, India

^2^ Department of Computer Science and Engineering, Netaji Subhash Engineering College, Garia, Kolkata, India

^3^ Department of Computer Science and Engineering, Jadavpur University, Kolkata, West Bengal, India

^4^ Centre of New Technologies, University of Warsaw, Warsaw, Poland

^5^ Faculty of Mathematics and Information Science, Warsaw University of Technology, Warsaw, Poland

Corresponding Author:

Dariusz Plewczynski ^4,5^

[Banacha 2c](https://maps.google.com/?q=Banacha+2c&entry=gmail&source=g) Street, 02-097 Warsaw, Poland

Email address: d.plewczynski@cent.uw.edu.pl

# DEFINITIONS AND NOTATIONS

Before proceeding into the main section of our work, it is important to discuss the graphical properties as well as other relevant terms associated with our work.

**Protein-protein interaction network:** Protein-protein interactions occur when two or more proteins bind together, often to carry out their biological function. These protein interactions form a network like structure which is known as a protein interaction network. Protein interaction network is generally represented as a graph consisting of a set of nodes connected by edges or links. Proteins are represented as nodes in the graph and the edges signify interactions between two proteins. Here protein interaction network is represented as a graph $G_{p}$which consists of a set of vertex V (nodes) connected by edges E (links). Thus$G=\left( V \right.,\left. E \right)$.

**Protein complex/cluster:** It can be defined as [group](http://www.businessdictionary.com/definition/group.html) of [proteins](http://www.businessdictionary.com/definition/server.html) (usually in close proximity to one another) interconnected through a [network](http://www.businessdictionary.com/definition/network.html) to [work](http://www.businessdictionary.com/definition/work.html) as one [centralized](http://www.businessdictionary.com/definition/centralization.html) [data processing](http://www.businessdictionary.com/definition/data-processing.html) resource. Here it is defined by $C_{i}$ where i represent cluster number.

**Sub graph:** A graph ${G'}_{p}$ is a sub graph of $G_{p}$ if the vertex set of ${G'}_{p}$ is a subset of the vertex set of $G_{p}$ and if the edge set of ${G'}_{p}$ is a subset of the edge set of  $G_{p}$. That is, if ${G´}_{p}=\left( V' \right.,\left. E' \right)$and$G_{p}=\left( V \right.,\left. E \right)$, then ${G´}_{p}$ is called as sub graph of $G_{p}$ if $V'\subseteq V$ and$E'\subseteq E$. ${G'}_{p}$  may be defined as a set of $\left\{ P_{U} | P_{A} \right\}$ where $P_{U}$ represents the set of un-annotated proteins while $P_{A}$ represents the set of annotated protein.

$\mathbf{Level-1}$ **Neighbors:** For any vertex $v$ in ${G'}_{p}$, all those vertices in ${G'}_{p}$that are connected with $v$ through an edge are deemed $Level-1$ neighbors of $v$.

**Edge weight (**$\mathbf{W}_{\mathbf{uv}}$**):** The weight $W_{\mathrm{uv}}$of edge $(u, v)$ (Wang & Wu 2013) is defined as the similarity between u and v. It is obvious that two nodes with an edge between them belong to the same cluster if they have high similarity. The similarity between u and v is measured by Jaccard’s coefficient. Jaccard’s coefficient adopts the proportion of common neighbors of two nodes in all distinct neighbors of these nodes to measure node similarity in complex networks. Obviously, the more common neighbors two nodes share, the higher similarity these nodes have. Therefore, the edge weight $W_{\mathrm{uv}}$ is represented by

where, Γ(u) and Γ(v) are neighbors of u and v respectively. Γ(u) ∩ Γ(v) represents all common neighbors of u and v, and Γ(u) ∪ Γ(v) represents all distinct neighbors of u and v. In our algorithm, edge weight is used to guarantee that in the same cluster every pair of nodes with an edge between them should have relatively high similarity.

**Neighborhood graph (*G_v_*):** The neighborhood graph of v ∈ V consists of v, all its neighbors and the edges among them. It is defined as *G_v_* = ($V', E'$), in which $V'$= {*v*} ∪ {*u*|*u* ∈ *V*, (*u*, *v*) ∈ *E}*, and $E'$= {(*u_i_*, *u_j_*)| (*u_i_*, *u_j_*) ∈ *E*, *u_i_*, *u_j_* ∈ $V'$}.

**Node weight**$\mathbf{(} \mathbf{W}_{\mathbf{v}}$**):** In $G_{v}$, there are some nodes with degree 1 that only have connections with v and the connections among these nodes are often false positive according to topological reliability measures (Wang & Wu 2013).So nodes with degree 1 and corresponding edges are removed from $G_{v}$. The remaining sub graph of $G_{v}$is marked as$G_{v}^{'}$.The node weight wv of node v ∈ $V$ in PPI networks is the average degree of all nodes in $G_{v}^{'}$. It is represented by

where, $V^{''}$is the set of nodes in $G_{v}^{'}$.|$V^{''}$| is the number of nodes in $G_{v}^{'}$. And deg(u) is the degree of a node $u\epsilon$ $V^{''}$in $W_{v}$. In our algorithm, the weight $W_{v}$ of a node v $\in$V is used in the step of seed chosen. Higher value of $W_{v}$ of a graph indicates a collection of nodes with maximum interactions among them and hence the graph is densely connected region.

**Physico-Chemical Properties (PCP):** Physico-Chemical Properties (Saha & Chatterjee 2014; Singh et al. 2008) of amino acids are the various features of protein which are used to predict protein class. These properties are very important in protein class prediction. The various Physico-Chemical Properties used in this work are as given below:

1. **Extinction Coefficient**$\mathbf{(}\mathbf{E}_{\mathbf{protein}}$**):** Extinction Coefficient (Singh et al. 2008) is a protein parameter that is commonly used in the laboratory for determining the protein concentration in a solution by spectrophotometry. It describes to what extent light is absorbed by the protein and depends upon the protein size and composition as well as the wavelength of the light. For proteins measured in water at wavelength of 280nm, the value of the Extinction coefficient can be determined from the composition of Tyrosine, Tryptophan and Cystine.

Mathematically it can be defined as:

$$\text{E}_{\text{protein}}\text{=(N}_{\text{tyr}}\text{×E}_{\text{tyr}}\text{)+(N}_{\text{trp}}\text{×E}_{\text{trp}}\text{)+(N}_{\text{cys}}\text{×E}_{\text{cys}}\text{)}$$

where $E_{\mathrm{tyr}}$ =1490, $E_{\mathrm{trp}}$ =5500, $E_{\mathrm{cys}}$ =125 are the Extinction coefficients of the individual amino acid residues.

1. **Absorbance (Optical Density):** For proteins measured in water at wavelength of 280nm the absorbance can be determined by the ratio of Extinction coefficient and the molecular weight of the protein. It is a representation of a material's light blocking ability (Singh et al. 2008).

Mathematically absorbance is defined as:

1. **Number of Negatively Charged Residues (**$\mathbf{N}_{\mathbf{neg}}$**):** This can be calculated from the composition of Aspartic acid and Glutamic acid (Singh et al. 2008).
2. **Number of Positively Charged Residues (**$\mathbf{N}_{\mathbf{pos}}$**):** This can be calculated from the composition of Arginine and Lysine (Singh et al. 2008).
3. **Aliphatic Index (AI):** The aliphatic index of a protein is defined as the relative volume occupied by aliphatic side chains (alanine, valine, isoleucine, and leucine). It may be regarded as a positive factor for the increase of thermo stability of globular (Singh et al. 2008).

Mathematically aliphatic index is defined as:

where $X_{\mathrm{ala}}$ , $X_{\mathrm{val}}$ , $X_{\mathrm{ile}}$ and $X_{\mathrm{leu}}$ are the mole percentages of alanine, valine, isoleucine and leucine respectively. Coefficients a and b are the relative volume of valine side chain and side chains to the side chain of alanine i.e. a = 2.9 and b = 3.9.

1. **Compute IP/Mol weight:** It calculates the isoelectric point by molecular weight (Singh et al. 2008) of the input amino acid sequence. IP stands for isoelectric point of the input amino acid sequence. Mol weight stands for molecular weight of the input amino acid sequence.
2. **Grand average of hydropathicity (GRAVY):** The GRAVY value for a protein or a peptide (Kyte & Doolittle 1982) is calculated by adding the hydropathy values of each amino acid residues and dividing by the number of residues in the sequence or length of the sequence. Increasing positive score indicates a greater hydrophobicity.
3. **Instability index:** The instability index (Guruprasad et al. 1990) provides an estimate of the stability of your protein in a test tube. A protein whose instability index is smaller than 40 is predicted as stable, a value above 40 predicts that the protein may be unstable.
4. **Aromaticity:** Aromaticity (Lobry & Gautier 1994) is simply the relative frequency of phenylalanine (Phe), tryptophan (Trp), tyrosine (Tyr).
5. **Isoelectric point:** The isoelectric point (Bjellqvist et al. 1994) is the pH at which a molecule or surface carries no net electrical charge.
6. $\mathbf{PCP}_{\mathbf{score}}$ **:** $\mathrm{PCP}_{\mathrm{score}}$ is defined as scaling of the mean value obtained from top-ranked physico-chemical properties among the properties mentioned above which are obtained by the execution of four classifiers: XGBoost classifier, Random Forest classifier, Extra Tree classifier and Recursive feature elimination classifier.

**XGBoost Classifier:** XGBoost is a scalable end to end tree boosting system which proves to be highly effective and widely used machine learning method for feature selection (Chen & Guestrin 2016; Pedregosa et al. 2011).

**Random forest Classifier:** A random forest is defined to be a meta-estimator that fits a number of decision tree classifiers on various sub-samples of the dataset and use averaging to improve the predictive accuracy and control over-fitting (Breiman 2001; Pedregosa et al. 2011).

**Extra Tree Classifier:** Extra Tree classifier is a new tree-based ensemble method for supervised classification of feature selection (Geurts et al. 2006; Pedregosa et al. 2011).

**Recursive feature elimination (RFE) Classifier**: RFE is used to select features by recursively considering smaller and smaller sets of features (Pedregosa et al. 2011). First, the estimator is trained on the initial set of features and the importance of each feature is obtained either through a *coef_* attribute or through a feature*_importances_* attribute. Then, the least important features are pruned from current set of features.

# INFORMATION REGARDING MIPS DATABASE

The PPIN of MIPS (Munich Information Center for Protein Sequences) (Mewes et al. 2002) database of yeast has been used in this work for considering protein pair along with its corresponding functions. This dataset is available at their website:

(ftp://ftpmips.helmholtzmuenchen.de/fungi/Saccharomycetes/CYGD/PPI/)

The Munich Information Center for Protein Sequences (MIPS-GSF, Neuherberg, Germany) (Mewes et al. 2002) continues to provide genome-related information in a systematic way. MIPS supports both national and European sequencing and functional analysis projects, develops and maintains automatically generated and manually annotated genome-specific databases, develops systematic classification schemes for the functional annotation of protein sequences, and provides tools for the comprehensive analysis of protein sequences. The MIPS dataset of yeast obtained from the link mentioned above contains protein pairs along with their corresponding functions like Protein A|Protein B|DNA Repair (suppose for example) i.e. when Protein A interacts with Protein B they perform the function DNA Repair (annotated). Mewes et al. (Mewes et al. 2002) stated these as “*Genomes that are being annotated and published by MIPS*”.

According to Mewes et al. (Mewes et al. 2002), as the amount of specialist yeast related data continues to grow, they are exploring a model to integrate additional data collections and knowledge into the Comprehensive Yeast Genome Database (CYGD). CYGD is built upon collaboration with several yeast laboratories and includes specialized databases. “*20000 newly identified genes*” from 13 hemiascomycetous yeasts, generated by the Genolevure project, have already been integrated. He also classified some of the genes to be “*Unfinished and/or unpublished genomic sequences*” in which he stated that “Gene prediction conducted by ORPHEUS in a completely automatic fashion, usually allows large overlaps between ORFs. This leads to many overpredicted ORFs, but ensures that *fewer real ORFs are missed*.”

This lead to the development of “two types of interactions” like this in their MIPS dataset:

1. Protein B|Protein C|unknown
2. Protein D|Protein E|missing

**Protein B|Protein C|unknown:** This signifies that Protein B interacts with Protein C. This interaction already exists but their implementing automated methodology for protein pair function prediction failed to predict the functions when Protein B interacts with Protein C. That’s why they have given “unknown” in the function field to signify that this is an existing pair whose function is yet to be predicted.

**Protein D|Protein E|missing:** This signifies that Protein D interacts with Protein E. But these are the newly identified proteins and interactions. While attempting to predict functional annotations in automated fashion this interaction gets missed due to excessive overlapping or overprediction of ORFs. That’s why they have given “missing” in the function field to signify that this is a missing interaction pair (newly predicted) whose function is yet to be predicted.

So both can be basically classified as “*unpredicted protein pair interactions*” i.e. protein interactions whose functional annotations are not yet predicted.

# INFORMATION REGARDING PROTEIN-PAIR FUNCTION PREDICTION

A protein may perform various functions in isolation. But it does not perform all the functions while reacting with another. It may perform some specific functions while interacting with one protein while perform some other specific functions while reacting with other proteins. So besides predicting protein function, protein pair function also needs to be determined. So various researches have been conducted in this field of study (Chatterjee et al. 2012; Shatsky et al. 2016). In disease based PPIN, where function of one protein (say Protein A) is known but the function of its interacting protein (responsible for causing disease)(say Protein B) is not known then function of Protein B can be predicted from Protein A since conventional approaches associate protein interaction with the sharing of functions: “if proteins A and B belong to the same functional pathway, A is likely to interact with B; therefore when A and B are observed to interact, they are likely to share functions” (Chua et al. 2006).

# INFORMATION REGARDING METHODOLOGY

FunPred 3 is broadly classified into two sections:

*First section* involves:

1. Selection of test set proteins (proteins considered as unannotated which are annotated in real).
2. Prediction of functional annotations of test set by the proposed methodology.
3. Computation of the effectiveness of the prediction of our proposed methodology through the computation of precision, recall and F-Score.

*Second section* involves only the prediction of unknown/missing protein pair function by our proposed methodology.

Hence Precision, recall and F-score have been computed in the first section. Since originally the functions of the test proteins are annotated but we consider them to be unannotated, so after the predicting the functions of test set proteins we can match them with the original defined ones. If it matches then it is considered as true positives. Similarly False Positives etc. are also computed. Suppose, for example Protein A has originally DNA Repair function and it is included in our test set proteins. So we consider that the function of Protein A is unannotated and hence predict it by our proposed methodology. Now if our proposed methodology predicts the function of Protein A as DNA Repair then we consider it as a match with the original one (i.e. True Positive) else not.

Since our methodology FunPred 3 chooses only the essential proteins as test set proteins (by the application of node and edge weight) in the entire PPIN of yeast so it has been observed that these essential proteins belong to near about 155 diversified functional groups which is extensively large when compared to its predecessors FunPred 1 and FunPred 2.

Both GO and the MIPS functional catalogues are hierarchical. But MIPS contain certain common GO functions. Moreover we have not used FunCat id (like 11.06.03.01, 16 etc.) for function prediction. Instead we have used direct functions (like mRNA editing, transcription) of proteins i.e. if protein A is originally annotated to “mRNA editing” in MIPS dataset and our prediction model annotates it as “transcription” then it is not considered as true positive. True positive is considered only when our prediction model annotates it as “mRNA editing”. FunCat id is considered as one of our future work which is already in progress.

# REFERENCES

Bjellqvist B, Basse B, Olsen E, and Celis JE. 1994. Reference points for comparisons of two-dimensional maps of proteins from different human cell types defined in a pH scale where isoelectric points correlate with polypeptide compositions. *ELECTROPHORESIS* 15(1):529-539. 10.1002/elps.1150150171.

Breiman L. 2001. Random Forests. *Machine Learning* 45(1):5-32. 10.1023/a:1010933404324.

Chatterjee T, Chatterjee P, Basu S, Kundu M, and Nasipuri M. 2012. Protein function by minimum distance classifier from protein interaction network. In: 2012 International Conference on Communications, Devices and Intelligent Systems (CODIS).588-591.

Chen T, and Guestrin C. 2016. XGBoost: A Scalable Tree Boosting System. In: Proceedings of the 22nd ACM SIGKDD International Conference on Knowledge Discovery and Data Mining. San Francisco, California, USA: ACM.785-794.

Chua HN, Sung W-K, and Wong L. 2006. Exploiting indirect neighbours and topological weight to predict protein function from protein–protein interactions. *Bioinformatics* 22(13):1623-1630. 10.1093/bioinformatics/btl145.

Geurts P, Ernst D, and Wehenkel L. 2006. Extremely randomized trees. *Machine Learning* 63(1):3-42. 10.1007/s10994-006-6226-1.

Guruprasad K, Reddy BVB, and Pandit MW. 1990. Correlation between stability of a protein and its dipeptide composition: a novel approach for predicting in vivo stability of a protein from its primary sequence. *Protein Engineering, Design and Selection* 4(2):155-161. 10.1093/protein/4.2.155.

Kyte J, and Doolittle RF. 1982. A simple method for displaying the hydropathic character of a protein. *Journal of Molecular Biology* 157(1):105-132. <https://doi.org/10.1016/0022-2836(82)90515-0>.

Lobry JR, and Gautier C. 1994. Hydrophobicity, expressivity and aromaticity are the major trends of amino-acid usage in 999 Escherichia coli chromosome-encoded genes. *Nucleic Acids Research* 22(15):3174-3180.

Mewes HW, Frishman D, Güldener U, Mannhaupt G, Mayer K, Mokrejs M, Morgenstern B, Münsterkötter M, Rudd S, and Weil B. 2002. MIPS: a database for genomes and protein sequences. *Nucleic Acids Research* 30(1):31-34.

Pedregosa F, Varoquaux G, Gramfort A, Michel V, Thirion B, Grisel O, Blondel M, Prettenhofer P, Weiss R, Dubourg V, Vanderplas J, Passos A, Cournapeau D, Brucher M, Perrot M, and Duchesnay E. 2011. Scikit-learn: Machine Learning in Python. *J Mach Learn Res* 12(2825-2830.

Saha S, and Chatterjee P. 2014. Protein Function Prediction from Protein Interaction Network using Physico-Chemical Properties of Amino Acids. *International journal of pharmacy and biological sciences* 4(2):55-65.

Shatsky M, Allen S, Gold BL, Liu NL, Juba TR, Reveco SA, Elias DA, Prathapam R, He J, Yang W, Szakal ED, Liu H, Singer ME, Geller JT, Lam BR, Saini A, Trotter VV, Hall SC, Fisher SJ, Brenner SE, Chhabra SR, Hazen TC, Wall JD, Witkowska HE, Biggin MD, Chandonia J-M, and Butland G. 2016. Bacterial Interactomes: Interacting Protein Partners Share Similar Function and Are Validated in Independent Assays More Frequently Than Previously Reported. *Molecular & Cellular Proteomics : MCP* 15(5):1539-1555. 10.1074/mcp.M115.054692.

Singh M, Wadhwa PK, and Kaur S. 2008. Predicting Protein Function using Decision Tree. *World Academy of Science, Engineering and Technology* 2(3):300-303.

Wang S, and Wu F. 2013. Detecting overlapping protein complexes in PPI networks based on robustness. *Proteome Science* 11(Suppl 1):S18-S18. 10.1186/1477-5956-11-S1-S18.
